# Supplementary material for: CD36 maintains the gastric mucosa and associates with gastric disease
Source: Commun Biol. 2021 Nov 2;4:1247. doi: 10.1038/s42003-021-02765-z (PMC8563937; doi:10.1038/s42003-021-02765-z)
Supplement: Supplementary file 2 — Supplementary Information [file 42003_2021_2765_MOESM2_ESM.pdf]

Supplementary Information

CD36 maintains the gastric mucosa and associates with gastric disease

Jacome-Sosa et al.

Supplementary Tables and Figures

Supplementary Table 1. List of antibodies, source and dilution used

**Marker**	**Species**	**Source**	**Cat. No.**	**Dilution**
CD36	Goat	R&D Systems	AF2519	1:100
Doublecortin-like kinase DCLK1,	Rabbit	Abcam	ab109029	1:100
Ezrin	Mouse	Santa Cruz Biotechnology	sc-58758	1:100
Ghrelin	Rabbit	Phoenix Pharmaceuticals	H-03-31	1:100
Chromogranin A	Rabbit	Abcam	ab45179	1:100
Gastrin	Rabbit	BioGenex	AR019-5R	1:200
Fibronectin	Rabbit	Abcam	ab2413	1:250
Vascular endothelial growth factor B, VEGFB	Goat	Santa Cruz Biotechnology	sc-1876	1:100
GS-II Lectin, Alexa Fluor® 647 Conjugate		Molecular probes	L21416	1:1000
Ki67	Rabbit	Abcam	ab16667	1:400
Gastric intrinsic factor, GIF	Rabbit	gift from Dr. David Alpers, Washington University, St. Louis		1:500

Supplementary Table 2. List of primer sequences.

Gene	Description	Primer sequences
Agpat1	1-acylglycerol-3-phosphate O-acyltransferase 1 (lysophosphatidic acid acyltransferase, alpha)	**F:** TAAGATGGCCTTCTACAACGCC<br>**R:** CCATACAGGTATTTGACGTGGAG
ATP4B	ATPase, H+/K+ exchanging, beta polypeptide	**F:** ACCTTCCAGGATGATCCCCA<br>**R:** CCAGAGGGTTGCTGTAGTGG
Chga	Chromogranin A (Chga)	**F:** AGG GGA CAC CAA GGT GAT GA<br>**R:** AGC AGA TTC TGG TGT CGC AG
Col1a1	Collagen, type I, alpha 1	**F:** CGA TGG ATT CCC GTT CGA GT<br>**R:** CGA TCT CGT TGG ATC CCT GG
Fn1	Fibronectin	**F:** GCC ACC ATT ACT GGT CTG GA<br>**R:** GGA AGG GTA ACC AGT TGG GG
GIF	Cobalamin binding intrinsic factor or gastric intrinsic factor (GIF)	**F:** CATGACATCCTGGGGCCTTA<br>**R:** GCAACCCCTTCATCCAAAGG
GPAT4	Glycerol-3-phosphate acyltransferase 4	**F:** AGC TTG ATT GTC AAC CTC CTG<br>**R:** CCG TTG GTG TAG GGC TTG T
IL10	Interleukin 10	**F:** GGC GCT GTC ATC GAT TTC TCC CC<br>**R:** AGC TCT GTC TAG GTC CTG GAG TCC
IL6	Interleukin 6	**F:** AGC CAG AGT CCT TCA GAG AGA T<br>**R:** GCA CTA GGT TTG CCG AGT AGA T
Lamb1	Laminin B1	**F:** CGG CAG GAA ATG GAA GGG<br>**R:** CCA TAG GGC TAG GAC ACC AA
Lep	Leptin	**F:** GAG ACC CCT GTG TCG GTT C<br>**R:** CTG CGT GTG TGA AAT GTC ATT G
Ccl2	Chemokine (C-C motif) ligand 2 (Ccl2) or monocyte chemotactin protein 1 (MCP1)	**F:** AGC TCT CTC TTC CTC CAC CA<br>**R:** CTA CAG CTT CTT TGG GAC ACC T
Gast	Gastrin	**F:** ATA GCA GAC CTG TCC AAG AAG C<br>**R:** GCC AAA GTC CAT CCA TCC GT
Ghrl	Ghrelin	**F:** CCC CAG GCA TTC CAG GTC AT<br>**R:** CAA ACT GCA GAT GGT GCC TG
Cxcl2	Chemokine (C-X-C motif) ligand 2 or macrophage inflammatory protein 2 (MIP2)	**F:** CTG TCA ATG CCT GAA GAC C<br>**R:** CCG GGT GCT GTT TGT TTT
Muc5ac	Mucin 5, subtypes A and C, tracheobronchial/gastric	**F:** GTC CTG AGG GTA TGG TGC TT<br>**R:** ACA TGT GTT GGT GCA GTC AGT A
Pgc	Pepsinogen C	**F:** AGCTTTGATCAGGGTCCCCC<br>**R:** GGCCAGGGTCATACTTGTGG
PNPLA2	Patatin-like phospholipase domain containing 2 or adipose triglyceride lipase (ATGL)	**F:** AAC CGG ACT CAC ATC TAC GG<br>**R:** CAG CAG GCA GGG TCT TTA GTA G

S100a8	S100 calcium binding protein A8 (calgranulin A)	**F:** GGA AAT CAC CAT GCC CTC TA<br>**R:** GCT ACT CCT TGT GGC TGT CTT T
Sst	Somatostatin	**F:** ACC CCA GAC TCC GTC AGT T<br>**R:** AAG TAC TTG GCC AGT TCC TGT T
Tff1	Trefoil factor 1	**F:** TAA ATT GTG GCT TCC CCG GT<br>**R:** AGG GAC ATT CTT CTT CTT GAG TGT
Tff2	Trefoil factor 2 (spasmolytic polypeptide)	**F:** CTG GTA GAG GGC GAG AAA CC<br>**R:** TGC TCC GAT TCT TGG TTT GGA
VEGFB	Vascular endothelial growth factor B	**F:** CTG ACG ATG GCC TGG AAT GT<br>**R:** TAT GGC AAC CCT GTC TGG CT

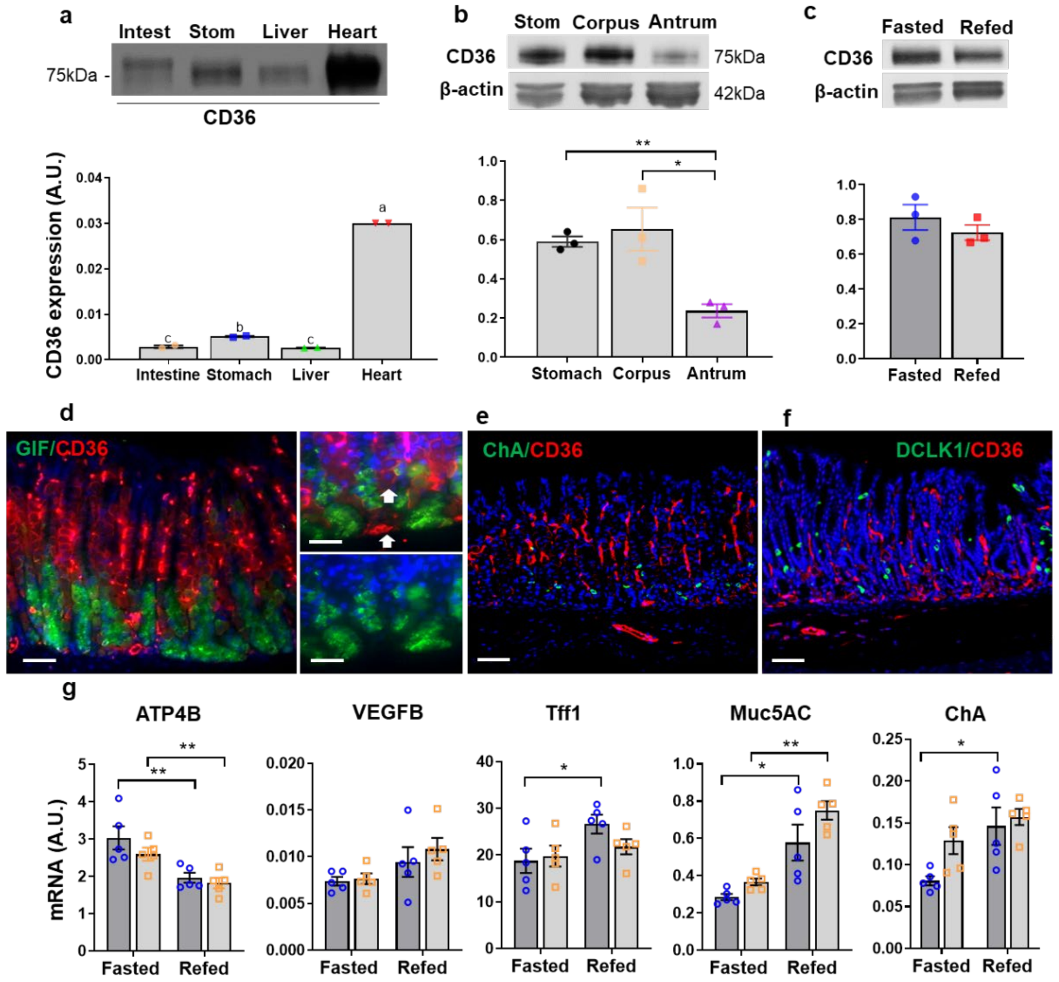

Supplementary Fig. 1: Stomach CD36 expression and gastric cell markers in germline *Cd36*$^{-/-}$ mice. a: CD36 in whole stomach (Stom), compared to proximal small intestine (intest), liver and heart (top) and adjusted to total protein (bottom). **b:** Expression in whole stomach, corpus and antrum adjusted to β-actin (bottom). **c:** Effect of fasting and refeeding, adjusted to β-actin (bottom). Immunostaining **d:** gastric intrinsic factor (GIF) (right insets: magnification of GIF+ cells showing lack of CD36 expression; white arrows: CD36+ parietal cells), **e:** chromogranin A (ChA), and **f:** Tuft cells expressing doublecortin-like kinase, DCLK1. Scale bar: 50 μm (**d, e, f**), and 20 μm (inserts in D). **g:** mRNA (qPCR) of cell markers for parietal cells: ATPase, H+/K+ exchanging, beta polypeptide (ATP4B), vascular endothelial growth factor B (VEGFB), for mucous cells: trefoil factor 1 (Tff1), spasmolytic polypeptide (Tff2), mucin 5AC (Muc5AC), and for enteroendocrine cells: ChA. Significance was calculated with a one-way ANOVA (**a-b**), two-sided unpaired t test (**c**) or a two-way ANOVA followed by *post hoc* tests and Sidak multiple comparisons (**g**). **P* < 0.05, ***P* < 0.01. Data are means ± SEM, n=2-3 for **a-c**, and n=5 for **g**.

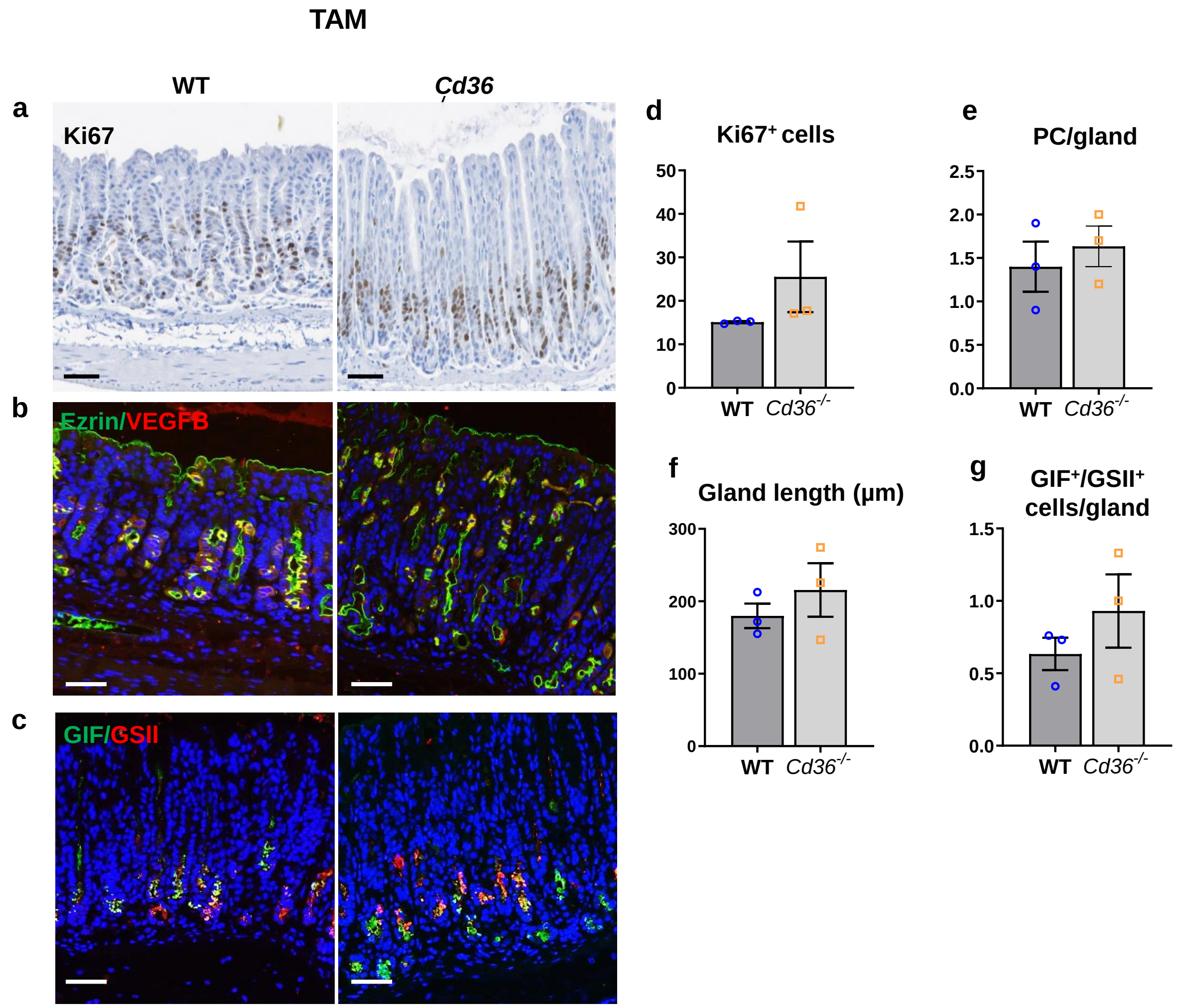

Supplementary Fig. 2: Tamoxifen (TAM) treatment induces parietal cell (PC) atrophy in wildtype (WT) and *Cd36*$^{-/-}$ mice. WT and *Cd36*$^{-/-}$ mice were injected with high-dose TAM for 3 days (TAM); Representative histology and immunohistochemistry: **a**: proliferation marker ki67; **b**: vascular endothelial growth factor B (VEGFB), red; ezrin, green; **c**: Gastric Intrinsic factor (GIF), green; Griffonia Simplicifolia Lectin II (GSII), red. Scale bar: 50 µm. Quantification of **d:** ki67^{+} cells, **e:** parietal cells per gastric unit, **f:** gland length, and **g:** GIF/GSII^{+} cells per gastric unit (yellow). Data were analyzed using a two-sided unpaired t test and are shown as means ± SEM, n=3 mice/group.

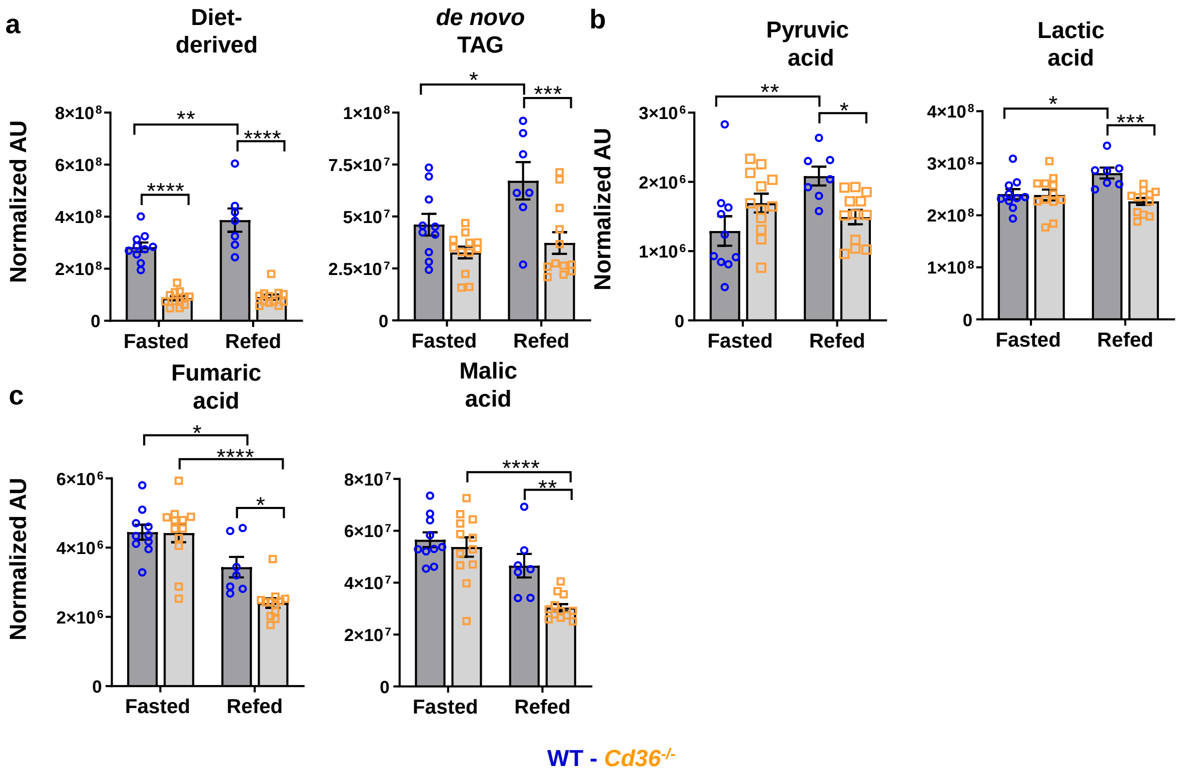

Supplementary Fig. 3: Metabolic alterations in *Cd36*$^{-/-}$ stomach. a: Lower levels of diet derived triglycerides, TAGs (combined 18:1 and 18:2) and de novo TAG species (combined 16:0, 16:1 with 14:0 or 12:0 fatty acids). Lower levels of **b:** glycolytic metabolites and **c:** Tricarboxylic acid (TCA) intermediates in *Cd36*$^{-/-}$ mice. Significance was calculated using a two-way ANOVA followed by *post hoc* tests by Sidak multiple comparisons. *P < 0.05, **P < 0.01, ***P < 0.001, ****P < 0.0001. Data are means ± SEM, n=7-12 mice/group.

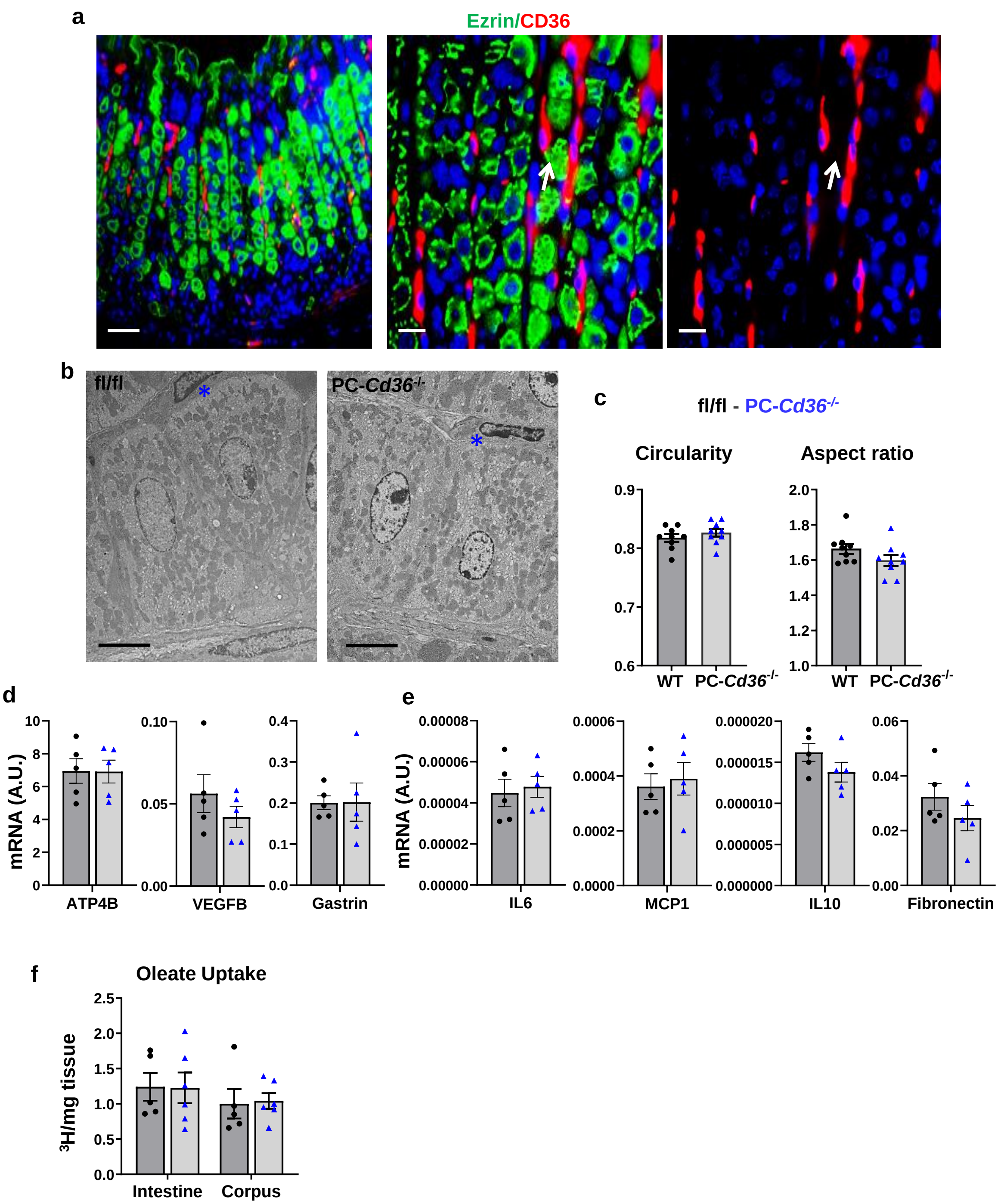

Supplementary Fig. 4: Deletion of *Cd36* in parietal cells (PCs) does not recapitulate stomach morphological abnormalities seen in *Cd36*$^{-/-}$ mice. PC-*Cd36*$^{-/-}$ mice were obtained by crossing *Cd36* floxed mice with mice carrying the ATP4b recombinase. **a:** Immunofluorescence of gastric sections from floxed controls and PC-*Cd36*$^{-/-}$ mice showing absence of CD36 expression on PCs (white arrow). Scales: 50 µm (first), 10 µm (second and third) panels. **b:** TEM showing

lack of mitochondria (“M”)-free area near basolateral plasma membrane of PCs from PC-*Cd36*$^{-/-}$ mice (right panel, blue asterisks). Scale: 6 µm**.** **c:** Quantification of mitochondrial circularity and aspect ratio (major/minor axis). **d:** mRNA of PC markers, of gastrin, and **e:** Fibronectin and inflammatory genes in PC-*Cd36*$^{-/-}$ mice. **f:** Stomach uptake of oleic acid is unchanged in PC-*Cd36*$^{-/-}$ mice. Mice (4-month) fasted for 16 h were given [^{3}H]oleic acid retro-orbitally and tissues collected at 5 min after injection for radioactivity measurements. Significance was calculated using a two-sided unpaired t test. Data are means ± SEM. **c**: n=1500 mitochondria from 9 PCs/genotype; **d-e**: n=5; **f:** n=5-6.

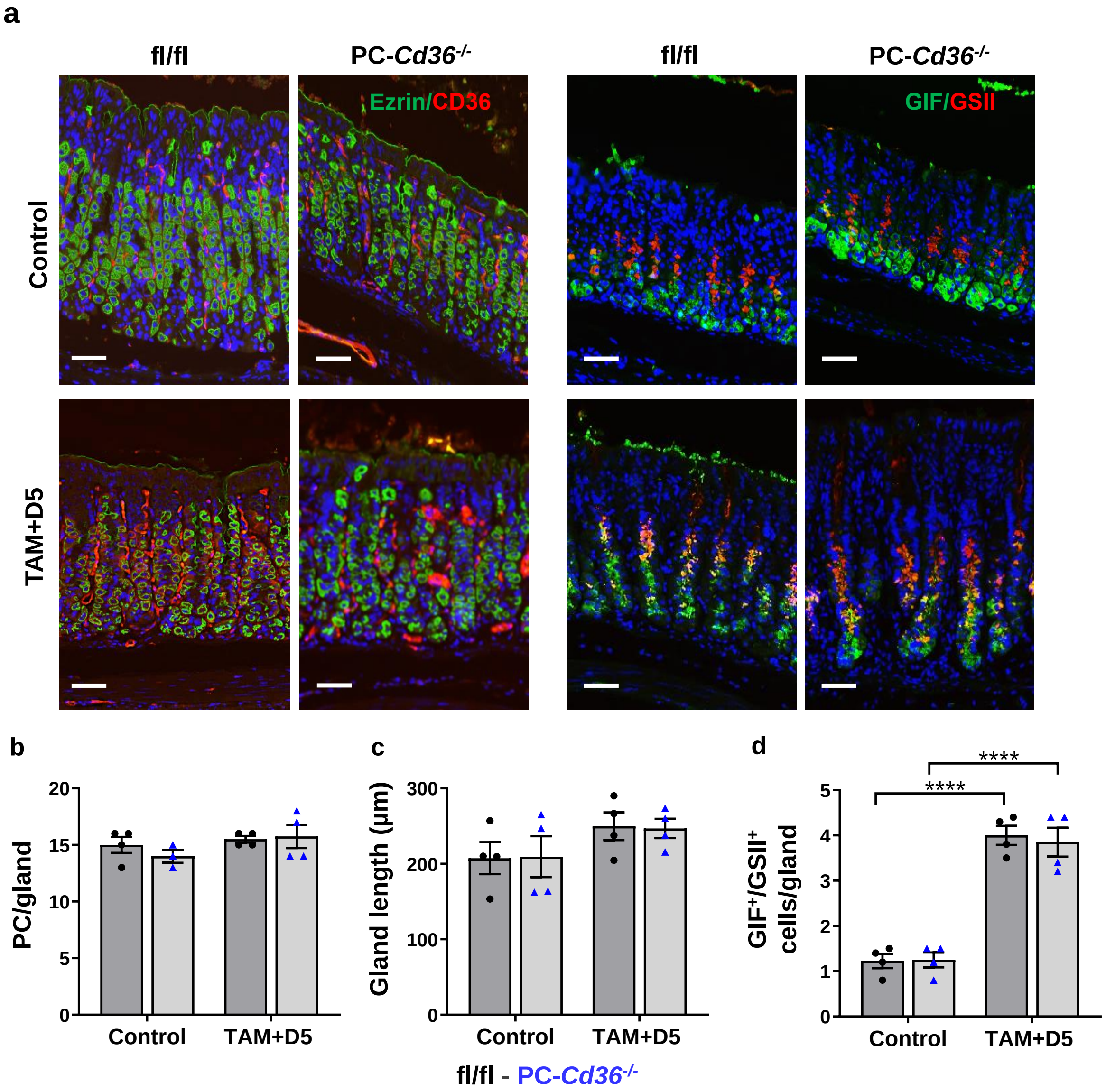

Supplementary Fig. 5: Deletion of *Cd36* in parietal cells does not alter PC renewal and recovery during gastric injury. a: Representative histology and immunohistochemistry of tissues of control and PC-*Cd36*$^{-/-}$ mice that were untreated (control) or injected with TAM for 3 days followed by 5 days recovery (TAM+D5), (*left*: ezrin, green; CD36, red; *right*: GIF, green; GSII, red). Scale bar: 50 µm**.** Quantification of **b:** Parietal cells per gastric unit, **c:** Gland length and **d:** GIF/GSII^{+} cells (yellow). Significance was calculated using a two-way ANOVA followed by *post hoc* tests by Sidak multiple comparisons. ****P < 0.0001. Shown are means ± SEM, n=4 mice/group.

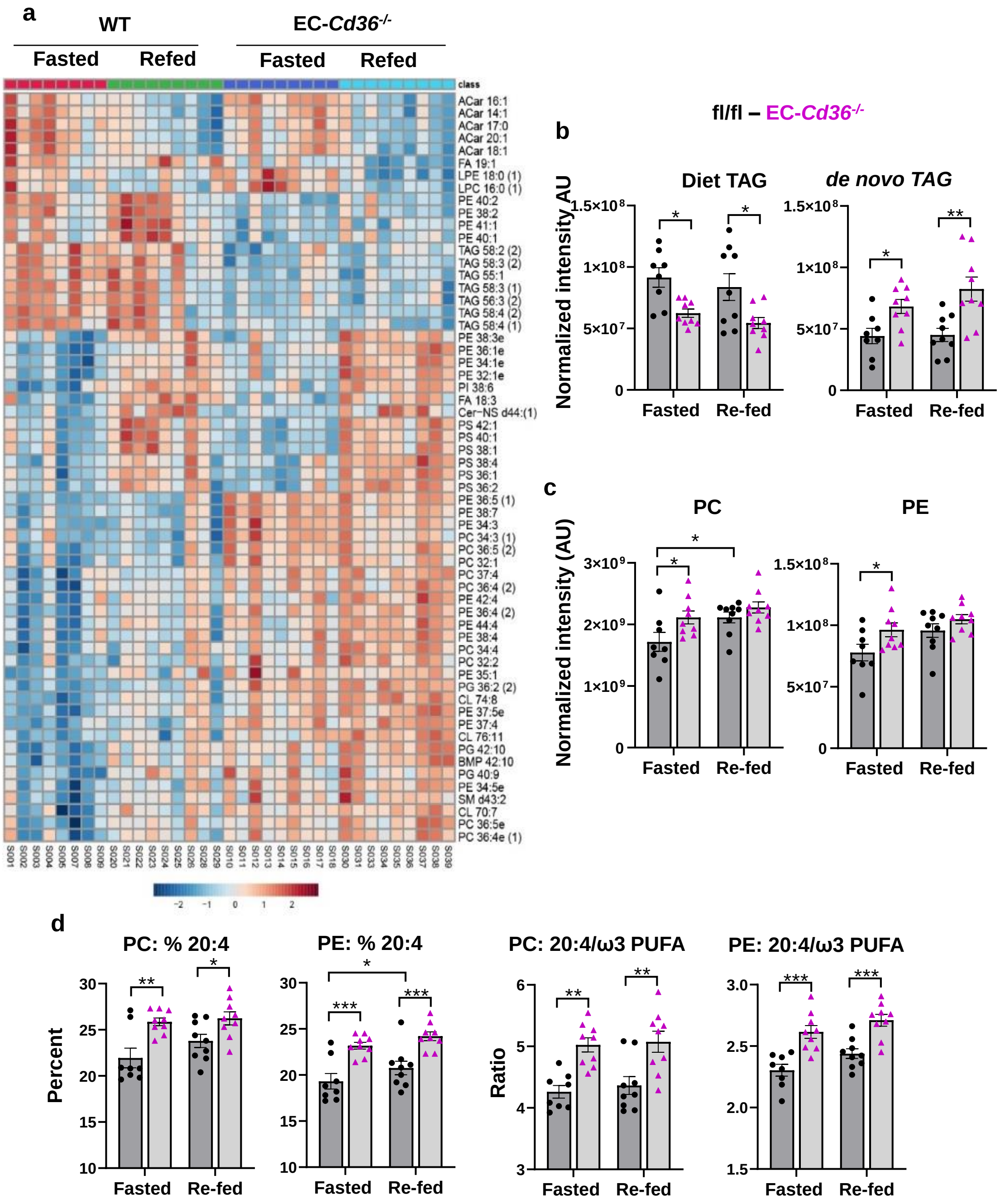

Supplementary Fig. 6: Altered lipid metabolism in EC-*Cd36*$^{-/-}$ stomach. a: Heat map of top 60 metabolites/lipids in fasted and refed control and EC- *Cd36*$^{-/-}$ stomachs. **b:** TAG species derived from dietary lipids (combination of 18:1 and 18:2) decreased while de novo TAGs (combination of 16:0, 16:1 with 14:0 or 12:0 fatty acids) were higher in EC- *Cd36*$^{-/-}$ mice. **c:** EC-*Cd36*$^{-/-}$ stomachs accumulate phospholipids with **d:** higher % 20:4 and greater ratio of 20:4 (ω6) to ω3 PUFA. PL, phospholipids; PC, phosphatidylcholine; PE, phosphatidylethanolamine; FA,

fatty acids; PUFA, polyunsaturated FA; TAG, triacylglycerol. Significance was calculated using a two-way ANOVA followed by *post hoc* tests by Sidak multiple comparisons. *$P < 0.05$, **$P < 0.01$, ***$P < 0.001$. Data are means ± SEM, n=8-9 mice/group.
