# Supplementary material for: CD36 maintains the gastric mucosa and associates with gastric disease
Source: Commun Biol. 2021 Nov 2;4:1247. doi: 10.1038/s42003-021-02765-z (PMC8563937; doi:10.1038/s42003-021-02765-z)
Supplement: Supplementary file 3 — Description of Additional Supplementary Files [file 42003_2021_2765_MOESM3_ESM.pdf]

Description of Additional Supplementary Files

File name: Supplementary Data 1

Description: Supplementary Data file shows the data used directly for generating Figures 1-7. Uncropped blot of Figure 2 is included. Uncropped blots of supplementary Figure 1a-c with MW markers have been included.
