# Supplementary material for: CD36 maintains the gastric mucosa and associates with gastric disease
Source: Commun Biol. 2021 Nov 2;4:1247. doi: 10.1038/s42003-021-02765-z (PMC8563937; doi:10.1038/s42003-021-02765-z)
Supplement: Supplementary file 5 — Reporting Summary [file 42003_2021_2765_MOESM5_ESM.pdf]

nature research

Corresponding author(s): Miriam Jacome-Sosa, Nada A Abumrad, Jason C Mills

Last updated by author(s): Oct 1, 2021

Reporting Summary

Nature Research wishes to improve the reproducibility of the work that we publish. This form provides structure for consistency and transparency in reporting. For further information on Nature Research policies, see our Editorial Policies and the Editorial Policy Checklist.

Statistics

For all statistical analyses, confirm that the following items are present in the figure legend, table legend, main text, or Methods section.

n/a	Confirmed	
☐	☒	The exact sample size (*n*) for each experimental group/condition, given as a discrete number and unit of measurement
☒	☐	A statement on whether measurements were taken from distinct samples or whether the same sample was measured repeatedly
☐	☒	The statistical test(s) used AND whether they are one- or two-sided<br>*Only common tests should be described solely by name; describe more complex techniques in the Methods section.*
☒	☐	A description of all covariates tested
☐	☒	A description of any assumptions or corrections, such as tests of normality and adjustment for multiple comparisons
☐	☒	A full description of the statistical parameters including central tendency (e.g. means) or other basic estimates (e.g. regression coefficient) AND variation (e.g. standard deviation) or associated estimates of uncertainty (e.g. confidence intervals)
☐	☒	For null hypothesis testing, the test statistic (e.g. *F*, *t*, *r*) with confidence intervals, effect sizes, degrees of freedom and *P* value noted<br>*Give P values as exact values whenever suitable.*
☒	☐	For Bayesian analysis, information on the choice of priors and Markov chain Monte Carlo settings
☒	☐	For hierarchical and complex designs, identification of the appropriate level for tests and full reporting of outcomes
☒	☐	Estimates of effect sizes (e.g. Cohen's *d*, Pearson's *r*), indicating how they were calculated

Our web collection on statistics for biologists contains articles on many of the points above.

Software and code

Policy information about availability of computer code

Data collection	Zeiss AxioVision LE and NDPScan software were used to collect images. ImageStudioLite 5.2 (Li-COR Biosciences) and QuantStudio Real-Time PCR software were used for collection of western blot images and qPCR data, respectively. Data mining used to explore CD36 mRNA associations with gastric disorders was conducted using the PrediXcan (https://github.com/hakyimlab/PrediXcan).
Data analysis	Statistical analysis was conducted using Graph Pad Prism 9. Metabolomic and lipidomic data were analyzed using MetaboAnalyst 4.0. Cell number, gland length, mitochondria size and shape were quantified using NIH ImageJ/FIJI. Photoshop was used to overlay images. The overlay of the CD36 gene (Fig. 8B) that illustrates the position of SNP rs144921258 in the gene was created in the UCSC Genome Browser (hg18) (https://genome.ucsc.edu/).

For manuscripts utilizing custom algorithms or software that are central to the research but not yet described in published literature, software must be made available to editors and reviewers. We strongly encourage code deposition in a community repository (e.g. GitHub). See the Nature Research guidelines for submitting code & software for further information.

Data

Policy information about availability of data

All manuscripts must include a data availability statement. This statement should provide the following information, where applicable:

- Accession codes, unique identifiers, or web links for publicly available datasets
- A list of figures that have associated raw data
- A description of any restrictions on data availability

Source data files are provided with this paper and all relevant data can be obtained from the corresponding authors Miriam Jacome-Sosa or Nada Abumrad upon reasonable request.

Field-specific reporting

Please select the one below that is the best fit for your research. If you are not sure, read the appropriate sections before making your selection.

- [x] Life sciences
- [ ] Behavioural & social sciences
- [ ] Ecological, evolutionary & environmental sciences

For a reference copy of the document with all sections, see nature.com/documents/nr-reporting-summary-flat.pdf

Life sciences study design

All studies must disclose on these points even when the disclosure is negative.

Sample size	For experiments involving quantification of cell proliferation and renewal after injury, n=3 was chosen as the minimal replicate number. Sample size was determined based on similar studies previously conducted by our group. We confirmed this to be sufficient based on internal control (specific staining of known markers) and low observed variability between samples.
Data exclusions	Data were not excluded from analysis.
Replication	To ensure robust reproducibility, all data presented in this manuscript were quantified from at least three biological replicates. Technical replicates were consistent among them (CV less than 5-10%). All images presented were imaged at least three times. For quantification of injury and recovery, at least 5 images with 10 gastric units were obtained by stomach and cells per gland were averaged for all units counted.
Randomization	For imaging, whole stomachs were processed with transverse stomach rings cut from the mid-corpus region and both greater and lesser curvature sampled. Sections used for imaging were randomly selected. Whole stomachs were used for qPCR and metabolomic and lipidomic analyses, with no sub-sampling and thus, no requirement for randomization.
Blinding	No blinding was performed in the experimental design as conditions were evident. However, the quantification of injury and recovery was performed by a research assistant who was blinded related to genotype and experimental conditions. Samples were identified by the mouse ID numbers.

Reporting for specific materials, systems and methods

We require information from authors about some types of materials, experimental systems and methods used in many studies. Here, indicate whether each material, system or method listed is relevant to your study. If you are not sure if a list item applies to your research, read the appropriate section before selecting a response.

Materials & experimental systems

n/a	Involved in the study	
[ ]	[x]	Antibodies
[x]	[ ]	Eukaryotic cell lines
[x]	[ ]	Palaeontology and archaeology
[ ]	[x]	Animals and other organisms
[x]	[ ]	Human research participants
[x]	[ ]	Clinical data
[x]	[ ]	Dual use research of concern

Methods

n/a	Involved in the study	
[x]	[ ]	ChIP-seq
[x]	[ ]	Flow cytometry
[x]	[ ]	MRI-based neuroimaging

Antibodies

Antibodies used	Goat anti-CD36 (R&D Systems, AF2519, 1:100), Rabbit anti-DCLK1 (Abcam, ab109029, 1:100), Mouse anti-Ezrin (Santa Cruz Biotechnology, sc-58758, 1:100), Rabbit anti-Ghrelin (Phoenix Pharmaceuticals, H-03-31, 1:100), Rabbit anti-Chromogranin A (Abcam,a b45179,1:100), Rabbit anti-Gastrin (BioGenex, AR019-5R, 1:200), Rabbit anti-Fibronectin (Abcam, ab2413, 1:250), Goat anti-VEGFB (Santa Cruz Biotechnology, sc-1876, 1:100), GS-II Lectin, Alexa Fluor 647 and 594 (Molecular Probes, L21416, 1:1000), Rabbit anti-Ki67 (Abcam, ab16667, 1:400), Rabbit anti-Intrinsic factor (gift from Dr. Alpers, Washington University, St. Louis, 1:500), Donkey anti-Goat, -Rabbit, -Mouse Alexa Fluor 488, 647 and 594 (Invitrogen, 1:500).
Validation	All antibodies used for immunohistochemistry have been validated by the previous studies conducted by the authors who routinely use them and the references are included. In addition, antibodies were validated in this study in knockout animal models for specificity. Antibodies for specific cell markers were validated by identifying the signal on the correct cell type.

Animals and other organisms

Policy information about studies involving animals; ARRIVE guidelines recommended for reporting animal research

Laboratory animals	All studies used cohorts of adult C57Bl6 mice, 12-20 week, littermates matched for age and sex.
Wild animals	The study did not involve wild animals.
Field-collected samples	The study did not involve samples collected from the field.
Ethics oversight	The mouse work was performed under the study protocol approved by the Washington University Institutional Animal Care and Use Committee.

Note that full information on the approval of the study protocol must also be provided in the manuscript.
